# Can Introns Stabilize Gene Duplication?

**DOI:** 10.3390/biology11060941

**Published:** 2022-06-20

**Authors:** Gioacchino Micheli, Giorgio Camilloni

**Affiliations:** 1 Istituto di Biologia e Patologia Molecolari CNR, Università Sapienza, P.le A. Moro 5, 00185 Roma, Italy; gioacchino.micheli@fondazione.uniroma1.it; 2 Dipartimento di Biologia e Biotecnologie “C. Darwin”, Università Sapienza, P.le A. Moro 5, 00185 Roma, Italy

**Keywords:** gene duplication, introns, genome evolution

## Abstract

**Simple Summary:**

Eukaryotic genes are organized as DNA sequences containing exon and intron regions. Exons relate to sequences that, after transcription, will be maintained in mature mRNA to provide the blueprint for protein translation. Introns, on the other hand, are present in the primary transcript and are then removed by the splicing mechanisms. The evolutionary solutions that maintain and make this complex gene organization functional are only partially known. Here, we speculate that the presence of introns in the gene sequence can stabilize the products of gene duplication, one of the most effective driving forces in genome evolution. The hypothesis we propose is to be considered additional to those currently reported and not as an alternative.

**Abstract:**

Gene duplication is considered one of the most important events that determine the evolution of genomes. However, the neo-duplication condition of a given gene is particularly unstable due to recombination events. Several mechanisms have been proposed to justify this step. In this “opinion article” we propose a role for intron sequences in stabilizing gene duplication by limiting and reducing the identity of the gene sequence between the two duplicated copies. A review of the topic and a detailed hypothesis are presented.

## 1. Essential Aspects of Gene Duplication

Several processes are surmised to drive the evolution of genomes. Among them, gene duplication has been traditionally regarded as a valuable source of innovation and functional variation, and its active involvement in genome evolution has been extensively considered [1,2,3]. The gene duplication rate is estimated in the same order of magnitude as single nucleotide polymorphisms [4].

**Figure 1:**
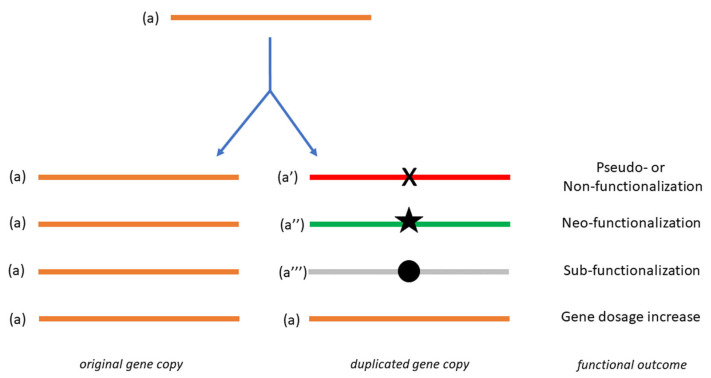
Schematic representation of gene duplication and its consequences. The symbols X, star and full circle denote one or more mutations which, after gene duplication, result in the functional outcomes specified on the right. The functional state of the duplicated gene is highlighted in color (orange, unchanged; red, non- or pseudofunctional; green, neo-functional; grey, sub-functional).

The concept of gene duplication was originally formalized by S. Ohno in 1970 [5] and its major features were successively highlighted by the same author [6]. In particular, Ohno considered that since only one gene copy is generally sufficient to support a given function, any extra copy obtained by duplication could undergo various mutations that could make it either non-functional (pseudo- or non-functionalization, Figure 1 a-a′), or cause the acquisition of a new function (neo-functionalization, Figure 1 a-a″). Moreover, if the second gene copy is not further modified, gene dosage would be increased [7] (Figure 1 a-a). Finally, through a specialization inducing an altered function, a sub-functionalization could occur [8] (Figure 1 a-a‴). According to Ohno’s model, there would be an overall increase in the evolutionary rate of the duplicated copy. Actually, at least two processes somehow limit the spread of new gene variants: accumulation of mutations, with the consequent formation of pseudogenes [2,9,10,11], and gene conversion, inducing homogenization with the original gene copy [12]. In essence, as gene duplication operates in all genomes [13] it allows evolution to experiment with otherwise prohibitive gene variants, and therefore, confers a strong evolutionary advantage [14].

Gene duplication events are observed both at a small scale (SSD) [15,16,17] and at a large scale, as exemplified by global genome duplication (WGD) [18,19]. SSD can derive mainly from tandem duplication events and unequal crossing over between paralogous genes. SSD usually involves genes placed in proximity to each other [15,16,17]. Transposition events can also lead to gene duplication and the traces of these events are observed in the conserved terminal repeats [20,21]. As for large-scale gene duplication, it is worth mentioning polyploidy (partial or temporary). This condition typically arises from alloploidy processes, the acquisition of related chromosomes from different species, autoploidy duplications caused by lack of cytokinesis, or fertilization between gametes that have not undergone a reduction of entire chromosomes [3]. The latter case is usually identified as WGD. Temporary changes in ploidy have been found in most organisms [3,22] and sequence analysis allows to identify the traces of these events. These phenomena are observed frequently in plants [23,24,25]. Generally, in WGD most duplicated genes are short-lived: one of the two copies is soon lost or altered and only one copy is eventually maintained. Interestingly, it has been observed that genes encoding products that operate in protein complexes tend to be maintained even as double copies [26]. The fact that in most cases of gene duplication only one copy is maintained has led to speculate that the initial “double copy” state is inherently unstable, the loss of one of the two copies being the result of unequal recombination.

Overall, what appears in current genomes can be regarded essentially as a balance between the acquisition and loss of gene copies. The widely acknowledged genetic relevance of gene duplication notwithstanding, the molecular mechanisms that can lead to the duplication of genes and to their initial stabilization are as yet not fully understood. In the “hypothesis” section we propose a role for intronic sequences precisely in the stabilization of the sequences of neo-duplicated genes.

## 2. Genomes and Non-Coding DNA Content

By comparing genome size in organisms along the phylogenetic scale a progressive, though not linear, increase can be observed starting with the simplest species. In bacteria and simple eukaryotes, there is a rather high gene density, while in the genomes of more complex eukaryotes gene density decreases with the increasing amount of DNA. These observations have led to the formulation of the well-known C-value paradox [27,28]. In essence, on the evolutionary scale, the size of the genome and the number of genes do not grow in parallel. The expansion of repeated copies, together with polyploidization processes, global genome duplications, and insertion of transposons, are major factors responsible for the increase in the size of genomes. Due to both pseudogenization and sub-functionalization processes, a large part of the increased genome size does not produce new genes, and with subsequent duplications, it accumulates as “junk DNA”. However, maintaining this pool of non-coding sequences allows for the acquisition of new biological functions that can represent a reservoir for biological evolution [29,30].

In this context, it is worth stressing that according to the genotype-phenotype dualism [31] the content of genomic DNA (genome size) represents an adaptive feature that changes during evolution just like a phenotypic trait. Hence, deciphering how genomes grow and how the formation/loss of genes and junk DNA occurs is crucial to the understanding of genome evolution

## 3. Introns

A very important genomic feature, which also provides a conspicuous contribution to the size of eukaryotic genomes, is the presence of introns, originally also called intervening sequences. They are found in most eukaryotic genes. The long-standing “one gene, one polypeptide chain” paradigm [32] suddenly became much less stringent after Sharp and colleagues discovered introns about forty years ago [33,34]. Initially (and for a long time) considered “junk DNA”, introns have been gradually recognized as having an increasingly important role in gene expression.

Introns are currently classified into three major categories according to their structure and the way in which they are removed from precursor RNA to produce its mature form [35]: (a) spliceosomal introns, which are ubiquitous in eukaryotic genomes and require a complex RNA/protein machinery (the spliceosome) for their removal from the RNA precursor; (b) self-splicing introns (ribozymes), subdivided into group I (present in bacteria, viruses and, in eukaryotes, in the rRNA fraction of mitochondria and plastids; their splicing involves a complex three-dimensional structure and the formation of lariats) and group II (found in bacteria, mitochondria, and chloroplasts; their excision involves a specific secondary structure of the precursor RNA); and (c) tRNA introns.

Almost four decades after their discovery, the debate on the origin of introns still remains open [36]. In particular, a hot topic is the early- versus late-appearance of these sequences during evolution. In the early-appearance hypothesis, introns are regarded as very ancient elements which, depending on the organism, have been successively lost in different ways [37]. Bacteria would have lost almost all of them in a streamlining process of the genome, while eukaryotes, particularly those endowed with large genomes, would have preserved intronic sequences in large quantities [38]. The late-appearance view is supported by the strong similarity between self-splicing group II introns and spliceosomal introns (which are surmised to derive from group II ones, as suggested by the formation of lariat structures in both systems and by the conservation of boundary sequences [39]). In essence, the group II autosplicing form is regarded as the original one which then largely evolved into spliceosomal introns.

Since “introgenesis” has continued during the course of evolution [40], current views assume the coexistence of the two intron origin processes, so that a pool of early-appearing introns is maintained with late-appearing ones that continue to accumulate. Regardless of the mechanisms that allowed the presence of introns, their maintenance must be subject to natural selection, and therefore to the existence of a functional advantage. Several specific advantages can be envisaged. For instance, an increased coding repertoire resulting from alternative splicing (AS) could represent an evolutionary “push” towards the creation of isoforms (giving rise to possible variants and new biological functions [41]), or towards the introduction of slight changes allowing the fine-tuning of specific functions [42] or the appearance of non-functioning forms [43,44]. Another advantage is represented by the possible increase of homologous recombination between similar sequences (exon shuffling) [35,45]. Finally, there is the possibility for faster evolution compared to coding sequences, as introns are not blocked by the need to specify coding information [46].

## 4. Hypothesis

We wish to suggest a further advantage for coding sequences in eukaryotic genomes to acquire or retain intervening sequences. The hypothesis we propose should be considered as an addition and not as an alternative to those summarized above. In particular, we focus on tandem-based duplications, as these exemplify a major gene duplication category.

Let us first consider the “intron early-appearance” scenario, i.e an initial genomic condition characterized by introns present in most genes. When gene duplication occurs, the maintenance of two or more identical copies is difficult due to purge systems of the genome, e.g., unequal homologous recombination which tends to restore the single-gene condition and to control repeated sequences, as it occurs (in a specialized form) for ribosomal genes. The presence of the intron(s) contributes to the rapid differentiation of one gene copy from the other. Indeed, as intronic sequences are non-coding they can mutate easily. A rapid progressive reduction in the homology between the two genes can be expected and, as sequence homology is the basis for efficient recombination, this, in turn, decreases the possibility for recombination to occur. Hence, the duplicate copy is quickly allowed to maintain its differentiated state.

Let us now consider the “intron late-appearance” scenario, i.e. a genomic condition characterized by the absence of introns in most genes. Following gene duplication, the acquisition of intervening sequences within the genes significantly lowers the identity between the sequences and minimizes the possibility of recombination that would lead to the loss of a copy. The acquisition of other introns further reduces the progress of this process and increases the stability of the sequence. In line with this expectation, exon size would exhibit minimal changes while intron size would be hypervariable. By reducing the homology between the gene copies, the acquisition of new introns thus stabilizes their presence.

In essence, both acquisition and maintenance of intervening sequences would allow (at least in the case of tandem duplications) the targeted gene to undergo duplication with a lower risk of being eliminated by homologous recombination. The association of introns with gene duplication enhances the stability of the duplicated copies and both processes are evolutionarily supported.

## Data Availability

Not applicable.

## References

[1] Vosseberg J., van Hooff J.J.E., Marcet-Houben M., van Vlimmeren A., van Wijk L.M., Gabaldón T., Snel B. (2021). Timing the origin of eukaryotic cellular complexity with ancient duplications. Nat. Ecol. Evol..

[2] Carvalho C.M.B., Zhang F., Lupski J.R. (2010). Genomic disorders: A window into human gene and genome evolution. Proc. Natl. Acad. Sci. USA.

[3] Kuzmin E., Taylor J.S., Boone C. (2021). Retention of duplicated genes in evolution. Trends Genet..

[4] Lynch M., Conery J.S. (2000). The Evolutionary Fate and Consequences of Duplicate Genes. Science.

[5] Ohno S. (1970). Evolution by Gene Duplication.

[6] Ohno S. (1999). Gene duplication and the uniqueness of vertebrate genomes circa 1970–1999. Semin. Cell Dev. Biol..

[7] Kondrashov F.A., Kondrashov A.S. (2006). Role of selection in fixation of gene duplications. J. Theor. Biol..

[8] Conrad B., Antonarakis S.E. (2007). Gene Duplication: A Drive for Phenotypic Diversity and Cause of Human Disease. Annu. Rev. Genom. Hum. Genet..

[9] Innan H. (2009). Population genetic models of duplicated genes. Genetica.

[10] Kimura M., King J.L. (1979). Fixation of a deleterious allele at one of two “duplicate” loci by mutation pressure and random drift. Proc. Natl. Acad. Sci. USA.

[11] Li W.H. (1980). Rate of gene silencing at duplicate loci: A theoretical study and interpretation of data from tetraploid fishes. Genetics.

[12] Liao D., Pavelitz T., Kidd J.R., Kidd K.K., Weiner A.M. (1997). Concerted evolution of the tandemly repeated genes encoding human U2 snRNA (the RNU2 locus) involves rapid intrachromosomal homogenization and rare interchromosomal gene conversion. EMBO J..

[13] Kondrashov F.A., Rogozin I.B., Wolf Y.I., Koonin E.V. (2002). Selection in the evolution of gene duplications. Genome Biol..

[14] Innan H., Kondrashov F. (2010). The evolution of gene duplications: Classifying and distinguishing between models. Nat. Rev. Genet..

[15] Taylor J.H., Woods P.S., Hughes W.L. (1957). The Organization and Duplication of Chromosomes as Revealed by Autoradiographic Studies Using Tritium-Labeled Thymidinee. Proc. Natl. Acad. Sci. USA.

[16] Smithies O. (1964). Chromosomal Rearrangements and Protein Structure. Cold Spring Harb. Symp. Quant. Biol..

[17] Koszul R., Caburet S., Dujon B., Fischer G. (2004). Eucaryotic genome evolution through the spontaneous duplication of large chromosomal segments. EMBO J..

[18] Kellis M., Birren B.W., Lander E.S. (2004). Proof and evolutionary analysis of ancient genome duplication in the yeast Saccharomyces cerevisiae. Nature.

[19] Wolfe K.H., Shields D.C. (1997). Molecular evidence for an ancient duplication of the entire yeast genome. Nature.

[20] Hughes A.L., Friedman R., Ekollu V., Rose J.R. (2003). Non-random association of transposable elements with duplicated genomic blocks in Arabidopsis thaliana. Mol. Phylogenet. Evol..

[21] Zdobnov E.M., von Mering C., Letunic I., Bork P. (2005). Consistency of genome-based methods in measuring Metazoan evolution. FEBS Lett..

[22] Byrne K.P., Wolfe K.H. (2005). The Yeast Gene Order Browser: Combining curated homology and syntenic context reveals gene fate in polyploid species. Genome Res..

[23] Jaillon O., Aury J.-M., Noel B., Policriti A., Clepet C., Casagrande A., Choisne N., Aubourg S., Vitulo N., Jubin C. (2007). The grapevine genome sequence suggests ancestral hexaploidization in major angiosperm phyla. Nature.

[24] Tuskan G.A., Difazio S., Jansson S., Bohlmann J., Grigoriev I., Hellsten U., Putnam N., Ralph S., Rombauts S., Salamov A. (2006). The genome of black cottonwood, Populus trichocarpa (Torr. & Gray). Science.

[25] Sémon M., Wolfe K.H. (2008). Preferential subfunctionalization of slow-evolving genes after allopolyploidization in Xenopus laevis. Proc. Natl. Acad. Sci. USA.

[26] Papp B., Pál C., Hurst L.D. (2003). Dosage sensitivity and the evolution of gene families in yeast. Nature.

[27] Thomas C.A. (1971). The genetic organization of chromosomes. Annu. Rev. Genet..

[28] Cavalier-Smith T. (1978). Nuclear volume control by nucleoskeletal DNA, selection for cell volume and cell growth rate, and the solution of the DNA c-value paradox. J. Cell Sci..

[29] Adelman K., Egan E. (2017). Non-coding RNA: More uses for genomic junk. Nature.

[30] Bernardi G. (2019). The Genomic Code: A Pervasive Encoding/Molding of Chromatin Structures and a Solution of the “Non-Coding DNA” Mystery. Bioessays.

[31] Bolondi A., Caldarelli F., Di Felice F., Durano D., Germani G., Michetti L., Tramutolo A., Micheli G., Camilloni G. (2017). What is a Gene? A Two Sided View. Evol. Biol..

[32] Beadle G.W., Tatum E.L. (1945). Neurospora. II. Methods of Producing and Detecting Mutations Concerned with Nutritional Requirements. Am. J. Bot..

[33] Berget S.M., Moore C., Sharp P.A. (1977). Spliced segments at the 5’ terminus of adenovirus 2 late mRNA. Proc. Natl. Acad. Sci. USA.

[34] Chow L.T., Gelinas R.E., Broker T.R., Roberts R.J. (1977). An amazing sequence arrangement at the 5’ ends of adenovirus 2 messenger RNA. Cell.

[35] Irimia M., Roy S.W. (2014). Origin of Spliceosomal Introns and Alternative Splicing. Cold Spring Harb. Perspect. Biol..

[36] Collins R.A., Stajich J.E., Field D.J., Olive J.E., DeAbreu D.M. (2015). The low information content of Neurospora splicing signals: Implications for RNA splicing and intron origin. RNA.

[37] Cavalier-Smith T. (1991). Intron phylogeny: A new hypothesis. Trends Genet..

[38] Cavalier-Smith T. (1985). Selfish DNA and the origin of introns. Nature.

[39] Lambowitz A.M., Zimmerly S. (2011). Group II introns: Mobile ribozymes that invade DNA. Cold Spring Harb. Perspect. Biol..

[40] Koonin E.V. (2006). The origin of introns and their role in eukaryogenesis: A compromise solution to the introns-early versus introns-late debate?. Biol. Direct.

[41] Gabut M., Samavarchi-Tehrani P., Wang X., Slobodeniuc V., O’Hanlon D., Sung H.-K., Alvarez M., Talukder S., Pan Q., Mazzoni E.O. (2011). An alternative splicing switch regulates embryonic stem cell pluripotency and reprogramming. Cell.

[42] Lopez A.J. (1998). Alternative splicing of pre-mRNA: Developmental consequences and mechanisms of regulation. Annu. Rev. Genet..

[43] Bingham P.M., Chou T.B., Mims I., Zachar Z. (1988). On/off regulation of gene expression at the level of splicing. Trends Genet..

[44] Yap K., Lim Z.Q., Khandelia P., Friedman B., Makeyev E.V. (2012). Coordinated regulation of neuronal mRNA steady-state levels through developmentally controlled intron retention. Genes Dev..

[45] Gilbert W. (1978). Why genes in pieces?. Nature.

[46] Chamary J.-V., Hurst L.D. (2004). Similar rates but different modes of sequence evolution in introns and at exonic silent sites in rodents: Evidence for selectively driven codon usage. Mol. Biol. Evol..

